# Tissue is an issue in the search for biomarkers in idiopathic pulmonary fibrosis

**DOI:** 10.1186/s13069-015-0020-2

**Published:** 2015-03-02

**Authors:** Riitta Kaarteenaho, Elisa Lappi-Blanco

**Affiliations:** Respiratory Research Unit, Medical Research Center Oulu, Oulu University Hospital, University of Oulu, Oulu, Finland; Respiratory Research Unit, Department of Internal Medicine, University of Oulu, Oulu, Finland; Unit of Medicine and Clinical Research, Pulmonary Division, University of Eastern Finland, Kuopio, Finland; Center for Medicine and Clinical Research, Division of Respiratory Medicine, Kuopio University Hospital, Kuopio, Finland; Department of Pathology, Oulu University Hospital, Oulu, Finland; Department of Pathology, University of Oulu, Oulu, Finland

**Keywords:** Usual interstitial pneumonia, UIP, Immunohistochemistry, Microarray, Proteomic, Transcriptomic

## Abstract

Biological markers, i.e., biomarkers, in lung tissue may make it possible to connect cell biological phenomena to the pathogenetic mechanisms in idiopathic pulmonary fibrosis (IPF). This review focuses on the lung tissue biomarkers, which have been compared with relevant clinical endpoints or with the most common differential diagnostic lung diseases. In addition, studies conducted on lung tissue samples and investigated by transcriptomic or proteomic methodologies have been included. Several studies have observed changes in alveolar epithelium and extracellular matrix supporting the current hypotheses of the pathogenesis of IPF. In many studies, however, alterations in inflammatory cells have been revealed, a phenomenon not currently incorporated into pathogenetic theories. Combining lung tissue material with other non-solid organs with clinically meaningful endpoints may prove to be the most beneficial approach in the search for non-invasive biomarkers.

## Review

### Introduction

Biological markers, which are often referred to as biomarkers, are commonly defined as objectively measured and evaluated indicators of physiological or pathological processes or pharmacological responses to therapeutic intervention [1], although there are also several other definitions. During recent years, blood-originated biomarkers from serum, plasma, or cells have been the most extensively reviewed with respect to idiopathic pulmonary fibrosis (IPF) [2,3]. Biomarkers have been postulated to be useful in several ways, e.g., in making a differential diagnosis between IPF and other interstitial lung diseases (ILDs), in estimating prognosis and survival, in revealing the course of disease, and also for monitoring drug efficacy. In addition, it is possible that biomarkers could be helpful in distinguishing between various phenotypes of IPF.

### Rationale for lung tissue biomarkers

It has been estimated that about one third of IPF patients do require a surgical lung biopsy (SLB) in order to come to an ultimate diagnosis, and thus, it may be feasible to obtain lung tissue samples from a relatively high proportion of patients [4]. One benefit for lung tissue biomarkers would be the fact that the tissue obtained is probably the most appropriate source if one wishes to be able to link cell biological phenomena to pathogenetic mechanisms of the disease. Many biomarkers can be presumably located in several targets. Blood, sputum, and even broncho-alveolar lavage (BAL) samples can be collected repeatedly, which is usually not possible for lung tissue samples taken by surgical operation, because these procedures always carry a potential risk of serious complications [4]. The novel less invasive method for obtaining lung tissue samples by the transbronchial cryo-biopsy technique is expected to become more common in clinical practice; this may mean that in the future, lung tissue samples could be obtained not only for diagnostics but also for follow-up [5]. Some blood biomarkers have been investigated also in BAL and lung tissue, but there are very few reports describing the simultaneous examination of blood and BAL or lung tissue samples. The recent study of Seibold et al. combined multiple sources of materials and showed that a polymorphism in the promoter of mucin-5 subtype B (MUC5B) was associated with familial interstitial pneumonia (IP) and IPF [6].

This review article aims to focus on biomarkers in IPF, i.e., idiopathic usual interstitial pneumonia (UIP), in lung tissue concentrating on studies with relevant clinical endpoints. Studies focusing on IPF and UIP were included due to the changes in the classification, which have taken place during the past decades, although all UIP cases do not necessarily represent IPF. Publications comparing IPF with major types of ILDs like nonspecific interstitial pneumonia (NSIP) and connective tissue disease-associated ILD (CTD-ILD), which are the most common differential diagnostic dilemmas, were included. In addition, studies conducted on lung tissue samples using modern large-scale transcriptomic and proteomic technologies were included, although all of those had not used clinical or radiological endpoints.

### Studies on lung tissue samples with clinical endpoints

#### Studies of fibroblast focus

The specific aggregates of fibroblasts, myofibroblasts, and extracellular matrix (ECM) proteins in fibrotic lung are called fibroblast foci (FF), and these structures are more common in IPF than in other types of lung fibroses. Several studies have demonstrated that a high amount of FF in lung tissue correlates with the shortened survival of IPF patients [7-12], as previously reviewed elsewhere [4]. At present, the number of the FF is the only histological biomarker that reproducibly correlates with the prognosis of IPF.

#### Lung tissue biomarkers with clinical or radiological endpoints

The majority, 75.8%, of patients with IPF were found to be positive for protease-activated receptor 2 (PAR-2) in the study of Park et al. (Table 1). Blood neutrophil counts were lower, whereas blood lymphocyte counts and honeycombing scores in chest CT were higher in PAR-2-positive patients than in the PAR-2-negative patients. All of the fatal cases belonged to the PAR-2-positive group, although this difference between groups did not reach statistical significance [13]. In the study of Tzouvelikis and co-authors, lung tissue samples of IPF, cryptogenic organizing pneumonia (COP), and NSIP patients were analyzed for epidermal growth factor receptor (EGFR). It was observed that the EGFR mRNA levels negatively correlated with forced vital capacity (FVC) and diffusion capacity (DLCO) [14].

**Table 1:** **Compilation of studies using lung tissue biomarkers with clinical endpoints**

**Study**	**Patients/sample type**	**Biomarker/method**	**Clinical endpoint**	**Main results**
[13]	33 IPF	PAR-2	Honeycombing in chest CT	Honeycombing, blood neutrophils, and lymphocytes increased in PAR-2-positive cases
			Inflammatory cells in blood and BAL	All fatal cases were PAR-2-positive
	SLB	IHC	All-cause mortality	
[14]	20 IPF	EGFR	Lung function	EGFR mRNA negatively correlated with FVC and DLCO
	15 COP	I collagen		
	15 NSIP			
	10 controls			
		IHC		
	SLB	qRT-PCR		
[15]	16 IPF	Inflammatory cells	Lung transplantation	Lymphocytes in lung tissue increased during progression
	SLB			
	Lung transplant	HC		
[16]	34 IPF	α-SMA	Survival	α-SMA and IL-4 associated with survival
		Telomerase		
		IL-4		
		TGF-β		
		β-FGF		
	SLB	IHC		
[17]	48 IPF	Serpin B3/B4	Cancer, dysplasia	High serpin B3/B4 associated with older age and impaired DLCO
		Ki-67	Age	
		TGF-β	Lung function	
	Lung transplant	IHC		
		RT-PCR		
[18]	29 IPF	Chymase	Change in FVC over 6-month period	Mast cell number associated slower rate decline in FVC in IPF
	16 HP	CAE		
	9 SSc-ILD	Eosinophil peroxidase		
	10 controls			
	SLB			
	Lung transplant	IHC		
[19]	19 UIP	SP-A	Prognosis	SP-A+ ratio lower in UIP cases who died
	17 NSIP	KL-6		
	1 COP			
	1 RB-ILD			
	4 IP			
	SLB	IHC		
[20]	24 UIP	Gremlin	Lung function	Gremlin mRNA correlates negatively with DLCO/VA and BMP-4 mRNA positively with FVC and DLCO
	12 NSIP	BMP-4	Differential diagnostics	
	SLB	IHC		Gremlin + area in IHC correlated negatively with FVC
	Lung transplant	RT-PCR		
[21]	20 IPF-UIP	CD68, anti-elastase, CD3, CD4, CD8	Lung function	FEV1 and survival correlated with CD3+ TLs, and CD68+ cells correlated with FEV1 in IPF
	20 AIP-DAD		Differential diagnostics Survival	
	20 NSIP			
	SLB	IHC		
[22]	12 IPF	CD68, anti-elastase, CD3, CD4, CD8	Lung function	CD8+ TLs correlated inversely with FVC% pred, TLC% pred, DLCO% pred, and PaO_2_
	SLB	IHC	PaO_2_	
			Dyspnea score	
[23]	IPF 29	LMP-1	Survival	LMP-1+ associated with death due to respiratory failure and use of systemic steroid
	SSc-ILD 5		Differential diagnostics	
	SLB	IHC	Systemic steroid treatment	
		PCR		
[24]	28 UIP	Tenascin-C	Survival	High tenascin-C expression correlated with shortened survival in UIP patients
	1 NSIP			
	6 DIP			
	6 sarcoidosis			
	6 BOOP			
	6 AA			
		IHC		
		IEM		
	SLB	Western blotting		

*IPF* idiopathic pulmonary fibrosis, *SLB* surgical lung biopsy, *IHC* immunohistochemistry, *CT* computed tomography, *PAR-2* protease-activated receptor 2, *BAL* broncho-alveolar lavage, *HC* histochemistry, *α-SMA* alpha smooth muscle actin, *IL-4* interleukin 4, *TGF-β* transforming growth factor-beta, *FGF* fibroblast growth factor, *qRT-PCR* quantitative reverse transcriptase polymerase chain reaction, *DLCO* diffusion capacity, *HP* hypersensitivity pneumonitis, *SSc-ILD* systemic sclerosis associated interstitial lung disease, *CAE* chloroacetate esterase, *UIP* usual interstitial pneumonia, *FVC* forced vital capacity, *NSIP* nonspecific interstitial pneumonia, *COP* cryptogenic organizing pneumonia, *BOOP* bronchiolitis obliterans organizing pneumonia, *RB-ILD* respiratory bronchiolitis interstitial lung disease, *SP-A* surfactant protein A, *KL-6* Krebs van den Lungen-6 antigen, *BMP-4* bone morphogenic protein 4, *AIP* acute interstitial pneumonia, *DAD* diffuse alveolar damage, *CD* cluster of differentiation, *FEV1* forced expiratory volume in 1 s, *PaO*_*2*_ arterial oxygen tension, *LMP-1* latent membrane protein 1, *DIP* desquamative interstitial pneumonia, *IEM* immunoelectron microscopy, *EGFR* epidermal growth factor receptor, *TLC* total lung capacity, *TL* T lymphocytes, *AA* allergic alveolitis.

Todd et al. studied IPF cases using both SLB and subsequent lung transplantation samples from each patient, which made it possible to compare the histological features of the early and late phases of the disease. It was revealed that numbers of lymphocytes in lung tissue increased during progression since the amount of lymphocytes was higher in the lung explants than in the SLB samples [15].

IPF cases were investigated for the expression levels of alpha smooth muscle actin (α-SMA), telomerase, interleukin 4 (IL-4), transforming growth factor-beta (TGF-β), and beta fibroblast growth factor (β-FGF). It was noted that the levels of expressions of myofibroblast α-SMA and IL-4 were negatively associated with patient survival [16]. Calabrese et al. examined explanted lungs of IPF patients, of which patients with high-grade dysplasia or carcinomas showed a greater increase in the levels of serpin B3/B4 expression in metaplastic epithelial cells than the patients without these diseases. The expression level of serpin B3/B4 was linearly and positively associated with age. Furthermore, the patients with greater impairments in DLCO displayed significantly higher expression of serpin B3/B4 [17].

It was noted that the number of mast cells were increased in IPF, and in addition, a high mast cell number also associated with a slower rate of decline in FVC in a study of Cha and co-authors [18]. Nagata and others evaluated Krebs von den Lungen-6 antigen (KL-6) and surfactant protein A (SP-A) in idiopathic interstitial pneumonia (IIP). In patients with IIPs as a whole and also in those with UIP, the SP-A positive ratio was significantly lower in those who died from the progression of disease in comparison to those patients with another prognosis, i.e., stable, improved, and deteriorating but living [19].

Myllärniemi et al. investigated UIP and NSIP cases for gremlin and bone morphogenetic protein 4 (BMP-4), revealing that the area of gremlin-positive staining correlated negatively with FVC. The levels of gremlin mRNA correlated negatively with the specific diffusion capacity corrected for alveolar volume (DLCO/VA), whereas BMP-4 mRNA correlated positively with FVC and DLCO [20]. A negative correlation between gremlin mRNA levels and DLCO/VA was observed when UIP and NSIP patients were analyzed. In contrast, a positive correlation was observed between BMP-4 mRNA and FVC as well as between BMP-4 mRNA and DLCO.

Parra et al. revealed that the total density of inflammatory cells was significantly increased in the patients with NSIP and diffuse alveolar damage (DAD) when compared to those with UIP. In UIP, forced expiratory volume in 1 s (FEV1) and survival correlated with the numbers of CD3-positive T lymphocytes (TL), the numbers of CD68-positive cells correlated with FEV1, and the amounts of neutrophil elastase-positive cells correlated with residual volume and residual volume/total lung capacity (TLC) and carbon monoxide transfer factor. The most important predictor of survival in UIP/IPF was CD3-positive TLs [21]. In another study it was found that in IPF, the numbers of CD8-positive TLs inversely correlated with FVC% predicted, TLC% predicted, DLCO% predicted, and arterial oxygen tension (PaO_2_). Positive and statistically significant correlations were found between the numbers of CD8-positive TLs and alveolar-arterial gradient (P(A-a)O_2_) as well as the Medical Research Council (MRC) score. Furthermore, the CD8-positive TLs displayed significant negative correlations with the FVC% predicted and the FEV1% predicted [22].

Tsukamoto et al. observed that epithelial cells were positively stained for Epstein-Barr virus-associated latent membrane protein 1 (LMP1) in 31% of the patients with IPF, whereas none of the patients with systemic sclerosis (SSc)-associated ILD or the controls showed this kind of positive staining. Death from respiratory failure was significantly more common in LMP1-positive patients than in LMP1-negative patients. The use of systemic steroids after lung biopsy was more frequent in the LMP-positive than in the LMP-negative patients [23].

The amount of tenascin-C was analyzed in patients with UIP and also other types of ILDs [24]. The mean survival of the patients with UIP with high scores of tenascin-C was significantly shorter than that of patients with UIP with a lower tenascin-C sum score. Testing of tenascin-C scores in different locations revealed that an increased accumulation of tenascin-C underneath metaplastic bronchiolar-type epithelium was also associated with a shorter survival.

#### Differential diagnostic biomarkers

Cipriani et al. investigated CTD-UIP and IPF to evaluate the count and area of both FF and lymphocyte aggregates (LAs) (Table 2). They found that FF counts and areas were lower in patients with CTD-UIP, whereas the LA counts and areas were greatest in the patients with CTD-UIP, although the differences did not quite reach statistical significance. The only marked difference was observed in NSIP features, which were more prevalent in CTD-UIP than in idiopathic UIP [25]. NSIP and IPF cases were evaluated for levels of chemokine receptors CXCR3 and CCR4. It was found that the number of CXCR3-positive lymphocytes in NSIP patients was significantly greater than the corresponding value in IPF patients. The number of CCR4-positive lymphocytes in NSIP patients was significantly lower than that in IPF, and thus, the CXCR3 to CCR4 ratio in the NSIP patients was significantly elevated [26].

**Table 2:** **Examples of studies focusing on differential diagnostics between IPF and NSIP or CTD-ILD**

**Study**	**Patients/sample type**	**Biomarker/method**	**Main results**
[25]	17 CTD-UIP	FF count/area	Lower FF counts/areas in CTD-UIP
	18 IPF-UIP	LA count/area	Higher LA counts/areas in RA-ILD
		NSIP features	Higher prevalence of NSIP in CTD-UIP
	SLB		
	Lung transplant	HC	
[26]	12 NSIP	CXCR3	CXCR3+ lymphocytes higher in NSIP
	10 IPF	CCR4	CCR4+ lymphocytes higher in IPF
	SLB	IHC	
[27]	9 IPF-UIP	Epimorphin	Epimorphin higher in NSIP than in IPF
	8 NSIP		
		IHC	
		Western blotting	
	SLB	Northern blotting	
[28]	8 IPF	p53, Mdm2, ubiquitin, Bax, p21	p53, phosphorylated p53, Mdm2 and Bax higher in IPF
	5 NSIP		
		IHC	
		TUNEL	
	SLB	Western blotting	
[29]	26 IPF-UIP	MMP-2, MMP-9, TIMP-2	More MMP-9 in IPF
	11 NSIP		More MMP-2 in NSIP and BOOP
	6 BOOP		
	SLB	IHC	

*CTD* connective tissue disease, *UIP* usual interstitial pneumonia, *IPF* idiopathic pulmonary fibrosis, *ILD* interstitial lung disease, *RA* rheumatoid arthritis, *SLB* surgical lung biopsy, *FF* fibroblast focus, *LA* lymphocyte aggregates, *NSIP* nonspecific interstitial pneumonia, *HC* histochemistry, *CXCR and CCR* chemokine receptors, *IHC* immunohistochemistry, *p53* tumor protein p53, *Mdm2* mouse double minute 2 homolog, *Bax* apoptosis regulator Bax, *p21* cyclin-dependent kinase inhibitor 1, *TUNEL* a method detecting DNA fragmentation resulting from apoptosis, *BOOP* bronchiolitis obliterans organizing pneumonia, *MMP* matrix metalloproteinase, *TIMP* specific tissue inhibitor of metalloproteinase.

The levels of epimorphin protein and mRNA expression in NSIP were significantly higher than those in the patients with UIP in the study of Terasaki and co-authors [27]. Nakashima et al. evaluated IPF and NSIP cases for signaling molecules associated with tumor protein p53-mediated apoptosis [28]. Western blotting revealed that the expression of p53, phosphorylated p53, and mouse double minute 2 homolog (Mdm2) protein was significantly higher in IPF and NSIP than in the controls. The numbers of cells positive for the p53, phosphorylated p53, Mdm2, and apoptosis regulator Bax proteins as well as the number of TUNEL-positive cells were higher in IPF than in NSIP. Suga et al. investigated cases with IPF, NSIP, and bronchiolitis obliterans organizing pneumonia (BOOP) for various matrix metalloproteinases (MMP) and the specific tissue inhibitors of metalloproteinases (TIMP) [29]. The intense expression of MMP-9 in metaplastic epithelial cells was a special characteristic of UIP.

#### Studies focusing on omics techniques

Zuo et al. conducted a microarray analysis of lung specimens from the patients with IPF and CTD-UIP (Table 3). A marked increase in the expression of genes that encode for muscle proteins was observed. The expression of genes that encode for proteins associated with cell contraction and actin filament organization was increased as well as that of genes encoding for collagens I, III and VI, tenascin-C, osteopontin, and fibronectin [30]. Selman et al. investigated lung tissues from the patients with IPF, hypersensitivity pneumonitis (HP), and NSIP by microarray [31]. IPF cases were enriched for genes involved in development, extracellular matrix structure and turnover, and cellular growth and differentiation. The levels of several epithelium-related genes were also upregulated in IPF lungs.

**Table 3:** **Omic studies using lung tissue in IPF research**

**Study**	**Patients/sample type**	**Method**	**Factors in IPF**
[30]	3 IPF-UIP	Oligonucleotide microarray	Muscle and smooth muscle markers, ECM proteins, factors associated with ECM formation, cell contraction, and actin filament organization increased in UIP
	2 CTD-UIP		
	3 controls from lung cancer surgery		
	5 controls from CLONTECH		
	SLB		
[31]	15 IPF	Affymetrix oligonucletide DNA microarray	Genes involved in development, epithelium, ECM structure/turnover, cellular growth, and differentiation upregulated in IPF
	12 HP		
	8 NSIP		
	Controls (trauma victims)		
	SLB		
[32]	14 IPF	Whole human genome oligonucleotide microarray	Factors involved in ECM turnover, structural constituents and degradation, and cell adhesion molecules increased in IIP
	2 NSIP		
	10 familial IIP (6 UIP, 4 NSIP)		
	9 normal controls		
	Autopsy		
	SLB		
	Lung transplant		
[33]	23 IPF (stable)	Agilent 4 × 4 whole human genome microarray	579 genes expressed differently in IPF and IPF-AEx: heat shock proteins, alpha-defensins, histones, and CCNA2
	8 IPF-AEx		
	12 control lung		
	Lung transplant		
	Autopsy		
[34]	6 stable or slowly progressive IPF	Serial analysis of gene expression (SAGE)	About 100 of transcripts upregulated in progressive IPF
	6 progressive IPF		
	SLB		
[35]	119 IPF-UIP	Affymetrix	Elevated expression of cilium genes associated with microscopic honeycombing, MUC5B, and MMP-7
	50 controls	Microarray	
	Lung tissue samples^a^	RT-PCR	
[36]	14 IPF	Proteome analysis: two-dimensional gel electrophoresis and MALDI-TOF-MS	51 upregulated and 38 downregulated proteins in IPF
	10 controls (donors)		
	Lung transplant	IHC	
[37]	IPF 14	2-dimensional DIGE technique and MALDI-TOF-MS	Stress-induced genes upregulated in IPF and fNSIP
	fNSIP 8		
	Controls 10 (organ donors)		
	Lung transplant		
[38]	8 IPF	Affymetrix oligonucleotide microarray	255 genes differentially expressed between IPF and controls
	8 PH-IPF		
	7 controls		
	Lung transplant		
	SLB		
[39]	40 IPF	Gene expression microarray	2,940 genes differentially expressed between IPF and controls
	8 controls	IHC	
	SLB		
[40]	94 IPF	Agilent gene expression microarray	2,130 DMRs of which 738 associated with changes in gene expression
	67 controls	CHARM array	
	Lung tissue samples^a^		

*UIP* usual interstitial pneumonia, *IPF* idiopathic pulmonary fibrosis, *CTD* connective tissue disease, *ECM* extracellular matrix, *HP* hypersensitivity pneumonitis, *NSIP* nonspecific interstitial pneumonia, *IIP* idiopathic interstitial pneumonia, *SLB* surgical lung biopsy, *AEx* acute exacerbation of IPF, *CCNA2* cyclin A2, *MUC5B* mucin-5 subtype B, *MMP* matrix metalloproteinase, *fNSIP* fibrotic nonspecific interstitial pneumonia, *DMR* differentially methylated regions, *IHC* immunohistochemistry, *RT-PCR* reverse transcriptase polymerase chain reaction.

^a^Lung tissue specimens (type not specified) obtained from the Lung Tissue Research Consortium.

Yang et al. profiled lung tissue from the patients with sporadic pulmonary fibrosis and patients with familial pulmonary fibrosis. The genes involved in ECM turnover, ECM structural constituents, proteins involved in ECM degradation, and cell adhesion molecules were increased. Most of the genes that were differentially expressed in the familial IIP belonged to the same functional categories as those that distinguished IIP from control samples, but they were over- or under-expressed to a greater extent in familial IIP than in all cases of IIP [32].

Konishi et al. evaluated lungs from the patients with stable IPF and patients with acute exacerbation of IPF (IPF-AEx) by microarrays. When compared with control samples, the global gene expression patterns of IPF-AEx were almost identical to those of stable IPF. In the direct comparison of IPF-AEx and stable IPF, the differentially expressed genes included those related to stress responses such as heat shock proteins and α-defensins as well as mitosis-related genes including histones and cyclin-A2 protein (CCNA2) [33]. The study of Boon and co-authors compared stable or slowly progressing IPF patients with those suffering from progressive IPF. It was found that about a 100 of transcripts were upregulated in the progressive group [34].

Yang et al. conducted a microarray analysis of 119 IPF cases and 50 controls. There was elevated expression of the cilium genes associated with microscopic honeycombing as well as higher expression of MUC5B and MMP-7. Two novel subtypes of IPF/UIP could be defined by the expression of cilium-associated genes [35]. Patients with high cilium gene expression demonstrated more microscopic honeycombing, but not FF, and displayed elevated tissue expression of MUC5B and MMP7.

A proteome analysis of explanted lungs from IPF patients identified that many proteins upregulated in the IPF fell into the related categories of unfolded protein response (UPR), endoplasmic reticulum (ER) stress, proteasome, degradation, and general cell stress response [36]. Subsequently, the same researchers evaluated IPF and fibrotic NSIP patients with proteomics and noted that the majority of the proteins which were upregulated in IPF and NSIP fell into the related categories such as chaperone/protein folding, protein processing, energy generation/glycolysis, and antioxidant function [37].

Patel et al. studied IPF patients with pulmonary hypertension (PH-IPF) and IPF patients without PH (NPH-IPF). The comparison of PH-IPF arteriole with NPH-IPF arteriole results achieved no separation between the two groups. When gene expression of the combined IPF samples was compared to the controls, a total of 255 genes were differentially expressed in IPF arterioles [38]. In a gene expression microarray study of DePianto et al., microscopic pathological heterogeneity in IPF lung tissue corresponded to patterns related to bronchiolization and lymphoid aggregates [39]. Recently, researchers were able to identify 2,130 differentially methylated regions in IPF, of which 738 were associated with significant changes in gene expression [40].

## Discussion

The current guidelines recommend that in the diagnostics of IPF, high resolution computed tomography (HRCT) has to be classified into three categories, i.e., 1) UIP, 2) probable UIP, or 3) not UIP [41]. If other diseases manifested as UIP are excluded, and if typical UIP is revealed in HRCT, then the diagnosis of IPF can be established on the basis of clinical and radiological investigations. When HRCT is categorized as possible UIP or not UIP, an examination of surgical lung biopsy is needed in order to make a reliable diagnosis, which is a multidisciplinary process and needs to be based on inputs from clinicians, radiologists, and pathologists [41].

The recent workshop on IPF emphasized three research foci, i.e., 1) alveolar injury, 2) cellular origins of myofibroblasts, and 3) role of stem/progenitor cells [42]. The present hypothesis of the pathogenesis of IPF indicates that injured and hyperplastic alveolar epithelia containing dysfunctional type II alveolar epithelial cells (AECII) are able to release factors causing proliferation of fibroblasts and myofibroblasts and deposition of ECM [43]. The pathogenesis of IPF resembles that of abnormal wound healing, a process involving many cell biological mechanisms including TGF-β and cellular stress [44]. The origin of the myofibroblasts in IPF is not yet totally clear, although several sources have been presented, e.g., that myofibroblasts arise from resident tissue fibroblast and bone marrow-derived cells, by epithelial mesenchymal transition (EMT) from epithelium, endothelium and pericytes, or from fibrocytes [43].

The levels of SP-A in alveolar epithelium have been shown to be lower in UIP patients dying from progressive disease in comparison with the patients with a stable disease, a finding which may reflect dysfunction of AECII [19]. The theory of epithelial damage has received further support from a study that used proteomics to reveal that many of the upregulated proteins in IPF belonged to the related categories of UPR and ER stress [36]. The finding in proteomics was also confirmed by immunohistochemistry highlighting that UPR markers were encountered in AECII cells in IPF but not in control cells [36]. The discoveries at the protein level are supported by a transcriptomic investigation, which showed that several epithelium-related genes were upregulated in IPF lungs [31]. A recent study has observed that the levels of cilium genes and MUC5B were increased in IPF and that cilium genes associated with microscopic honeycombing showing that not only alveolar but also bronchiolar epithelial alteration may have a role in the pathogenesis of IPF [35].

TGF-β-induced enhancement of ECM plays a fundamental role in the pathogenetic theories of IPF. Gremlin, an antagonist of BMP, which is a member of the TGF-β superfamily, has been shown to associate with lung function parameters in IPF [20]. High expressions of tenascin-C, an ECM protein, have been displayed to correlate with the shortened survival of the patients [24]. Surprisingly, few other immunohistochemical studies have been published on ECM alteration compared with clinical endpoints, whereas several studies have confirmed by transcriptomics that gene expression of various ECM proteins including collagens, tenascin, osteopontin, fibronectin, and genes involved in ECM regulation is upregulated in IPF [30-32]. The amount of α-SMA, which is a marker of myofibroblast, has been shown to associate negatively with patient survival in an immunohistochemical study [16], and an increased expression of the genes that code the factors associating with a myofibroblast has been presented by microarray studies [30,31]. Moreover, the high number of FF in lung tissue, in which myofibroblasts and ECM proteins are localized, has been shown to correlate with the shortened survival of the patients with IPF [4].

Many immunohistochemical studies have detected changes in the numbers of inflammatory cells in lung tissue of IPF, a finding which is not markedly supported by the current hypotheses. A recent study revealed that the amount of inflammatory cells did not decrease during the progression of the IPF as previously assumed by many [15]. Moreover, the number of mast cell has been shown to correlate with the progression of the disease [18], CD3-positive lymphocytes to correlate with FEV1 and survival [21], and CD68-positive T lymphocytes with lung function changes [22]. Furthermore, histological heterogeneity in IPF lung tissue corresponded to gene expression pattern related to lymphoid aggregates [39].

During the past decade, the research on IPF has focused on blood biomarkers, which is understandable since blood biomarkers can be easily obtained and analyzed, also repeatedly. At present, the most widely studied blood-derived biomarkers have been KL-6 and SP-A and -D [3]. As outlined in a recent review, a molecular biomarker in IPF should be quick, non-invasive, inexpensive, easy to repeat, and ideally blood or urine based [45]. Some blood biomarkers have been investigated also in BAL and lung tissue, but so far, there is a paucity of publications that have used simultaneously blood and BAL or lung tissue samples. It could be hypothesized, however, that the most reliable blood-derived biomarker could be found based on lung tissue analyses with the knowledge of cell-specific localization and with an established association with the course and the phenotype of the disease. In addition to the routine clinical follow-up investigations, it would be important to incorporate standardized staging and predictor models, such as GAP (gender, age, physiology) index, in any comparisons involving lung tissue biomarkers [46].

## Conclusions

Studying lung tissue in IPF is not an easy task when the missing key cell type or event in the pathogenesis of IPF causes discrepant analysis protocols in histological studies. Can we afford to ignore lung tissue samples when the pathogenesis of the disease and characterization of the phenotypes still leave so many unanswered questions? In addition to supplementing lung tissue material with other non-solid organs like blood, urine, and BAL, also the combination of multiple methods like omics, immunohistochemistry, protein assays, and mRNA quantification together with clinically meaningful endpoints may well prove to be the most beneficial approach with which to discover relevant non-invasive biomarkers. This kind of approach would hopefully make such progress so that within 10–15 years, rapid and reliable blood-based biomarkers will have become available not only for research purposes, but they will have become routine procedures also in clinical practice.

## Abbreviations

AECII: Alveolar epithelial type II cells
BAL: Broncho-alveolar lavage
Bax: Apoptosis regulator Bax also known as bcl-2 like protein 4
BMP-4: Bone morphogenetic protein 4
BOOP: Bronchiolitis obliterans organizing pneumonia
CCNA2: Gene encoding cyclin-A2 protein
CCR4: Chemokine receptor
CD: Cluster of differentiation
COP: Cryptogenic organizing pneumonia
CTD-ILD: Connective tissue disease-associated interstitial lung disease
CXCR3: Chemokine receptor
DAD: Diffuse alveolar damage
DLCO: Diffusion capacity
DLCO/VA: Specific diffusion capacity corrected for alveolar volume
ECM: Extracellular matrix
EGFR: Epidermal growth factor receptor
EMT: Epithelial mesenchymal transition
ER: Endoplasmic reticulum
FF: Fibroblast focus
FVC: Forced vital capacity
FEV1: Forced expiratory volume in 1 second
β-FGF: Beta fibroblast growth factor
GAP: Gender age, and physiology index
HP: Hypersensitivity pneumonitis
HRCT: high resolution computed tomography
IIP: Idiopathic interstitial pneumonia
IP: Interstitial pneumonia
ILD: Interstitial lung disease
IL-4: Interleukin 4
IPF: Idiopathic pulmonary fibrosis
IPF-AEx: Acute exacerbation of idiopathic pulmonary fibrosis
KL-6: Krebs von den Lungen-6 antigen
LA: Lymphocyte aggregate
LMP1: Latent membrane protein 1
Mdm2: Mouse double minute 2 homolog
MMP: Matrix metalloproteinase
MRC: Medical Research Council score
mRNA: Messenger ribonucleic acid
MUC5B: Mucin-5 subtype B
NPH-IPF: Idiopathic pulmonary hypertension patients without pulmonary hypertension
NSIP: Nonspecific interstitial pneumonia
P(A-a)O_2_: Alveolar-arterial gradient
PAR-2: Protease-activated receptor 2
PaO_2_: Arterial oxygen tension
PH-IPF: Idiopathic pulmonary hypertension patients with pulmonary hypertension
p21: Cyclin-dependent kinase inhibitor 1
p53: Tumor protein p53
RT-PCR: Reverse transcriptase polymerase chain reaction
qRT-PCR: Quantitative reverse transcriptase polymerase chain reaction
α-SMA: Alpha smooth muscle actin
SLB: Surgical lung biopsy
SP-A: Surfactant protein A
SP-D: Surfactant protein D
SSc-ILD: Systemic sclerosis associated interstitial lung disease
TGF-β: Transforming growth factor-beta
TIMP: Specific tissue inhibitor of metalloproteinase
TLC: Total lung capacity
TL: T lymphocytes
TUNEL: A method detecting DNA fragmentation resulting from apoptosis
UIP: Usual interstitial pneumonia
UPR: Unfolded protein response
